# Conceptualizing and Fostering the Quality of CES Through a Dutch National Network on CES (NEON)

**DOI:** 10.1007/s10730-020-09432-6

**Published:** 2021-01-15

**Authors:** Laura Hartman, Guy Widdershoven, Eva van Baarle, Froukje Weidema, Bert Molewijk

**Affiliations:** 1 Department of Medical Humanities, Amsterdam UMC, VU University Medical Centre, APH, Amsterdam, The Netherlands; 2 Netherlands Defense Academy, Breda, the Netherlands; 3 Centre for Medical Ethics, Institute of Health and Society, Faculty of Medicine, University of Oslo, Oslo, Norway

**Keywords:** Clinical ethics support, Quality, Responsive evaluation methodology, Learning

## Abstract

The prevalence of Clinical ethics support (CES) services is increasing. Yet, questions about what quality of CES entails and how to foster the quality of CES remain. This paper describes the development of a national network (NEON), which aimed to conceptualize and foster the quality of CES in the Netherlands simultaneously. Our methodology was inspired by a responsive evaluation approach which shares some of our key theoretical presuppositions of CES. A responsive evaluation methodology engages stakeholders in developing quality standards of a certain practice, instead of evaluating a practice by predefined standards. In this paper, we describe the relationship between our theoretical viewpoint on CES and a responsive evaluation methodology. Then we describe the development of the network (NEON) and focus on three activities that exemplify our approach. In the discussion, we reflect on the similarities and differences between our approach and other international initiatives focusing on the quality of CES.

## Introduction

Clinical ethics support (CES) aims to identify ethical questions in clinical practice and support health care professionals in dealing with these questions (Doran et al. 2016). Attention for CES is increasing and regulatory agencies which accredit health care institutions also increasingly stress the importance of having CES in a health care organization as part of quality of care and support for health care professionals (JCI 2018). Currently, CES comprises a range of different services, including ethics committees, ethics consultation, and moral case deliberation (MCD). These different services with varying aims often use a variety of methods and procedures (Molewijk et al. 2016). With the increase of CES services and CES innovations, the question about the quality of CES becomes more pressing: How can we conceptualize what a qualitatively good CES activity is and how can we foster the quality of CES?

In the Netherlands, a national network for clinical ethics support was established in 2014, called Netwerk EthiekOndersteuning Nederland (NEON). NEON aims to bring CES practitioners together in order to 1. learn from, collect and make best practice experiences regarding CES in healthcare organizations accessible; 2. professionalize CES by stimulating knowledge exchange and reflection on the quality of CES activities; and 3. conceptualize and foster the quality of CES by formulating a set of quality characteristics on CES. This paper focuses on the method we used to conceptualize and foster the quality of CES in the Netherlands. In another paper, we describe a specific outcome of this process, the quality characteristics of CES developed through and by NEON participants (Hartman et al. In Press).

In the following, we first describe activities of other national CES networks that have similar goals regarding fostering the quality of CES for their countries. We focus in particular on the methods these national networks use. In the second section, we outline the relationship between our theoretical viewpoint of CES and some key elements of a responsive evaluation methodology. In the third section, we describe three activities we undertook to conceptualize and foster the quality of CES and elaborate on the way they fit in with a responsive evaluation methodology. In the discussion section, we reflect on the similarities and differences between our methodological approach and other national networks set up to conceptualize and foster the quality of CES.

## National initiatives to conceptualize and foster the quality of CES

Internationally, there have been several initiatives to conceptualize and foster the quality of CES, approaching this endeavor from different angles. In this overview, we describe some exemplary national networks that were set up to conceptualize and foster the quality of CES for specific countries, presented in publications. We describe the initiatives of the American Society for Bioethics and Humanities (ASBH) in the USA, the UKCEN in the UK, and initiatives in Canada and Norway.^1^

The first set of standards and core competencies for ethics consultants was developed in 1998 in the United Sates by The Society for Health and Human Values-Society for Bioethics Consultation (SHHV-SBC 1998). To develop this document, 21 scholars participated in six “3-day meetings” over a period of two years, and they received comments on a draft from the “the bioethics community”.^2^ In 2006, The American Society for Bioethics and Humanities (ASBH) approved a motion to update this version and set up another taskforce (ASBH 2011).^9^ In 2009, a draft was again publicly available to comment on.^3^ The updated version of the *Core Competencies for Healthcare Ethics Consultation* identifies core skills, knowledge, and attributes for facilitating ethics consultations. These competencies are accompanied by five process standards of ethics consultations: open access, a systematic approach, a comprehensive policy and procedure, a notification protocol, and appropriate evaluations (ASBH 2011).

Since the publication of the *Core Competencies*, a tool has been developed to assess the quality of ethics consultations based on written records (Pearlman et al. 2016), an ethics code for individual health care ethics consultants has been published (Tarzian and Wocial 2015), and a document describing pearls and pitfalls of health care ethics consultants has been published (Carrese et al. 2012). Currently, the ASBH provides a certification program, the “Healthcare Ethics Consultant-Certified Program” that, “underscores the skills of health care consultants, with a national standard” (ASBH 2020c). As becomes clear from these varying initiatives, defining and fostering the quality of CES is approached from different angels ranging from defining core competencies, an ethics code to a certification program.

Simultaneously to the updates version of the *Core Competencies*, the National Center for Ethics in Health Care in the United States Government’s Department of Veteran Affairs (VA),^4^ developed the so called “IntegratedEthics program” (Fox et al. 2010). Both initiatives take each other’s work into account and reinforce each other (Magill 2013). The ASBH focuses on developing knowledge and skills for healthcare ethics consultants, the VA provides a comprehensive approach that describes a way to organize CES in a health care organization in such a way that CES is integrated throughout the whole health care organization. The VA initiative focuses specifically on how to organize CES in health care organizations when defining and fostering the quality of CES. The VA program describes three levels of CES functions that each target a specific level in a health care organization. First, ethics consultation deals with the level of decisions and actions; second, preventive ethics deals with the level of systems and processes; and third, ethical leadership deals with the level of environment and culture (Fox et al. 2010). To develop the IntegratedEthics program in-depth interviews were conducted and extensive input from internal and external stakeholders was obtained. Also, the design team evaluated the program through “validity testing, field testing, and a 12-month demonstration project in 25 separate health care facilities” (Fox et al. 2010, p. 2).

In the UK, a national network for clinical ethics committees (UKCEN) in hospitals has been set up (Slowther et al. 2004a, b). The goals of UKCEN are: “(1) To promote the development of ethics support in clinical practice in the UK, (2) To promote a high level of ethical debate in clinical practice and (3) To facilitate communication between all UK clinical ethics committees” (UKCEN 2020). The network has published a practical guide to ethics support (Anne Slowther et al. 2004a, b) and a set of core competencies for members of clinical ethics committees (Larcher et al. 2010). Also, through organizing conferences, a round robin and newsletters, the network facilitates communication between the clinical ethics committees. The website includes educational resourses for clinical ethics committee members on topics like genetics, vulnerable patient and the mental capacity act, a structured approach worksheet, contact details of clinical ethics committees, links to national guidelines on ethical discussions, and a discussion of hypothetical cases. To develop the core competencies an initial draft (inspired by the US document but modified for the UK context) was sent to the member committees of UKCEN for discussion and feedback. After redrafting and further discussion, a consensus was reached (Larcher et al. 2010).

In Canada, the network Practicing Healthcare Ethicists Exploring Professionalization (PHEEP) highlighted and reflected on several aspects of their activities in a special issue of the journal *HEC Forum* titled *Getting Engaged: Exploring Professionalization* (Volume 24, Issue 3).^5^ This included a methodology to develop professionalization-related “products”, such as practice standards for practicing health care ethicists (Kirby and Simpson 2012). A core group developed a decision-making framework for developing these products, based on deliberative engagement methodology that draws on social justice and deliberative democracy concepts. This product development framework consists of seven steps. Step one is establishing a core stakeholder working group and step two entails the collaborative consideration of “substantive values and principles” tailored to the specific product under development. In step three, relevant evidence and information is gathered to prepare for the fourth step: the development of product content through deliberative engagement.^6^ In the fifth step, an initial draft of the product is prepared, in which first, in step six, the feedback of the core stakeholders^7^ is incorporated and second, in the seventh step, the feedback of secondary stakeholders^8^ is incorporated. Through this methodology, the network was able to develop professionalization related products in an inclusive and democratic way, defining the quality of CES with both core stakeholders and secondary stakeholders (Kirby and Simpson 2012).

Finally, another example comes from Norway. In Norway every health care trust must have a clinical ethics committee. The Norwegian Ministry of Health and Care Services asked the Centre for Medical Ethics (CME) at the University of Oslo to coordinate the professional development of these clinical ethics committees. CME published a manual for working in a clinical ethics committee with detailed information about financing, meeting frequency, a six step model for case discussions, including a guide for the minutes of the meetings (Førde and Pedersen 2011; Pedersen et al. 2009a, b; Førde and Vandvik 2005; Pedersen et al. 2009a, b; Førde and Pedersen 2012a, b). The CME combined the development of the manual with research on the practice and quality of clinical ethics committees and on how it may be improved (Førde 2012). In addition, the CME created national projects and networks for CES in community health care in order to facilitate and study the quality of CES in community (Magelssen et al. 2016).

It becomes clear from these national networks that defining and fostering the quality of CES is approached from different angles and through a variety of methodologies. In the following, we describe our chosen methodology from a theoretical angle, explicating the connection between our chosen methodology and theoretical approach to CES. Then we describe three activities we undertook in more detail.

## The relationship between our theoretical understanding of CES and a responsive evaluation methodology to conceptualize and foster the quality of CES in the Netherlands

Our theoretical perspective on CES is inspired by hermeneutics and pragmatism, discourse or dialogical ethics, and ethics of care (Widdershoven and Molewijk 2010; Inguaggiato et al. 2019; Abma et al. 2010). These philosophical approaches emphasize that morality and ethics are experience based, contextual, dynamic and relational (Landeweer et al. 2017). This implies for CES that CES alone principally cannot “uncover” what is the objective or universally right thing to do for a moral issue. Instead, CES should be geared towards co-creating what is considered to be the moral good, together with stakeholders in a specific context. This requires facilitation and stimulation of moral inquiry in interaction with the stakeholders of the situation at hand. Various perspectives on a situation, entailing both practical experience and theoretical understanding, offer a relevant and potentially valid interpretation of the situation. CES should be focused on engaging these perspectives in a dialogue, fostering awareness of one’s own moral presuppositions and the differences with those of others leading to broadening of one’s horizon, and an enriched, more nuanced and shared understanding of a moral issue (Porz et al. 2011; Hartman et al. 2020).

A responsive evaluation methodology shares key presuppositions with our approach to CES (Hartman et. al 2020). A responsive evaluation methodology focuses on a joint reflection process with the involved stakeholders about both quality improvement of a certain practice and the criteria one uses to conceptualize and evaluate this quality (Abma et al. 2009). So instead of determining CES quality criteria beforehand or using an outsider’s expert-perspective and then measuring or finding ways to move a certain CES practice towards a predefined end goal, a responsive evaluation methodology takes the approach of including stakeholders and jointly evaluating and improving practices, while reflecting on the goals, definitions and criteria of the evaluation at the same time. Experiential knowledge is taken as a valid source of knowledge in this process, combined and refined by theoretical understanding. In this way, a co-construction of theoretical notions, experiences, values and narratives evolves (Guba and Lincoln 1989).

Responsive evaluation implies a cyclical process, in which all stakeholders are actively involved in the evaluation of the practices and the criteria on which the evaluation is based. Since the process needs to be flexible and responsive towards the views of stakeholders at a particular moment, the process needs to evolve along the way. In an emerging design, research findings are used as input for practice improvements, and practice experiences are used as input for the research process (Guba and Lincoln 1989; Abma and Widdershoven 2005; Stake 2004; Weidema 2014).

Conceptualizing and fostering the quality of CES in line with a responsive evaluation methodology implies involving stakeholders in CES in reflecting on and exchanging their views on the quality of their CES practices and the definitions and criteria they (often implicitly) use to determine this quality. CES practitioners often have an intuitive judgement about the quality of their own work. These judgments are usually implicit and dependent on one’s professional background, schooling, but also character and personal experiences, which together form one’s perspective. For instance, a CES practitioner educated in the four principles approach in bioethics will focus on these principles within a certain moral case and will assess the quality of a certain CES activity by whether or not the four principles are sufficiently taken into consideration. On the other hand, CES practitioners who are schooled in dialogical ethics will, in assessing the quality of a CES activity, focus on whether the process showed characteristics of a dialogue, that is a focus on listening and exchanging views, rather than presenting them in the form of discussion or debate.

In sum, a responsive evaluation methodology has similarities with our theoretical understanding of CES. First, both share the emphasis on dialogue between stakeholders. Second, both underline the importance of experiential knowledge and contextual details in the process of defining and fostering what is morally good or a good practice. Third, both aim not to produce abstract or general principles that will apply in all cases, but to assist stakeholders in reaching a fuller understanding of their practice or situation by including more perspectives and encouraging reflection on their (perhaps implicit) way of looking at a practice or case. Fourth, both view diversity or pluralism of ideas not as problematic but as an opportunity to learn and improve. Fifth, both focus on engagement with the practice under consideration instead of taking an outsider perspective that “objectively judges” a practice, and a commitment to the viewpoint that all voices should be heard and taken seriously. Applied to conceptualizing and fostering quality of CES, this means that we invite various stakeholders of CES to a dialogue about the (sometimes implicit or unarticulated) views on the quality of CES. The process entails both a reflection on the conceptualization of the quality of CES (i.e., what is actually a good quality CES activity?) and an improvement of the quality of CES. In the next section, we describe the development of NEON and three central activities we undertook to conceptualize and foster the quality of CES.

## Conceptualizing and fostering the quality of CES through NEON

In the Netherlands, CES services mainly consist of moral case deliberation (MCD) and ethics committees (Dauwerse et al. 2014). In Dutch hospitals, ethics committees are the most used type of CES. MCD is available in about half of Dutch hospitals and in two-thirds of the mental health care institutions (Dauwerse et al. 2014). Ethics consultants are sometimes available. Like in other countries, CES practitioners often feel isolated in their health care organization, trying to advocate the importance of (funding for) ethics support and trying to organize CES services by themselves or in a small team. The lack of shared knowledge and agreement about the quality of the various types of CES can lead to fragmentation and CES practitioners reinventing the wheel regarding CES services.

In order to meet the need of mutual exchange and support, NEON was established in 2014. NEON was founded in the context of a research project at VUmc (Amsterdam UMC) that started 1-12-2013, funded by the Ministry of Health of the Netherlands. When the research project ended (1-08-2018), the network continued as an independent foundation. Participants in NEON include about 260 individual CES practitioners working 160 Organizations. This includes members of ethics committees, facilitators of moral case deliberation, ethics consultants, managers of CES, academic ethicists, researchers, health care inspectors and policy makers in a variety of health care domains (e.g., hospital care, mental health care, care for the disabled, nursing care, youth care).

In the following, we describe three activities organized within NEON, focused on conceptualizing and fostering the quality of CES, and reflect on the way it fits with a responsive evaluation methodology.

### Organizing expert meetings and national conferences to facilitate a dialogue on quality of CES

To start encouraging joint reflection and knowledge exchange on CES, the researchers of the research project at VUmc (Amsterdam UMC) organized three expert meetings between 2013 and 2016. The meetings consisted of a core group, (about 25 people) of both CES theorists, CES practitioners in the Netherlands and other stakeholders (for instance, someone from the Dutch Inspectorate of Healthcare was present as well). The core group was selected through a snowball method and aimed to include a maximum of variety of CES stakeholders from different health care domains. In the meetings, the group was invited to formulate their views about the quality of CES and learn from each other. Two of the three meetings consisted of two days with a sleep–over to also stimulate informal relationships and get everybody away from their daily life. During the meetings the project group stimulated reflection and dialogue about the quality of CES, the goal of NEON and the desirability of developing quality characteristics for CES. Through the involvement from these CES stakeholders, we strived towards a feeling of co-ownership of both the process and the results of the project.

In the first expert meeting, (24–25 May 2014) a dialogue was facilitated on the quality of CES in a carrousel^9^ around the following three questions: 1. What are the goals of CES? 2. What is the right strategy for organizing CES? And, 3. what do you mean by quality of CES? Next, a carrousel was facilitated in which we asked: Towards which possible products should the network strive? The following products were mentioned: 1. a handbook for CES; 2. a website; 3. a network; 4. a guild group that develops standards for CES and education and training; and 5. scientific output. In the afternoon, both brainstorms were deepened and refined in subgroups.

In the second expert meeting (27 October 2014), reflection on quality of CES continued. Preliminary findings from 24 interviews with CES stakeholders were presented, in order to be discussed and deepened by the attendants. The interviews were about actual practices of CES, the quality of CES and the criteria the respondents used to determine this. Also, best practices regarding CES were shared by the participants of the meeting. The sharing of best practices was considered inspirational and a good way to reflect and foster the quality of CES. During the third expert meeting (26 and 27 March 2015), a website was launched (see Launch of a website and publishing a national handbook on CES), featuring among other things practice examples of CES. Also, the attendants were challenged to formulate statements about the quality of CES, followed by a dialogue about these statements. In the afternoon, the content of the different chapters of the Dutch handbook for ethics support was decided upon (see Launch of a website and publishing a national handbook on CES).

Besides these three expert meetings, national conferences were organized for everyone working with CES on either a theoretical of practical basis. The annual national NEON conferences started in 2014 and continued over the years. Like the expert meetings, the national conferences were geared towards joint refection processes, knowledge exchange, getting to know each other, quality improvement of CES and co-steering NEON. In the plenary lectures, influential and inspirational speakers were invited to reflect on the quality or importance of CES from a certain theme. In the workshops, the NEON participants themselves were asked to provide a workshop that they thought would be insightful for others. In this way, the participants of NEON trained each other. There was plenty of room in the program for networking, and peer-supervision on moral case deliberation and implementing CES.

The expert meetings functioned in three ways. Firstly, they provided insights into the perspectives and views of stakeholders of CES within the Netherlands regarding the quality of CES. Secondly, the stakeholders were challenged to make their implicit viewpoints on the quality of CES explicit. (This experience included our own research group who also were active during the meetings). For instance, a member of an ethics committee was asked what she considered to be the discerning expertise an ethicist brings to the ethics committee. These kinds of questions provoked the attendants to actively reflect and engage in a dialogue about the similarities and differences with other types of CES.

Third, challenges and doubts were discussed about the rational for and the form of the NEON quality characteristics. For example, does a formulation of quality characteristics of CES lead to exclusion of certain CES practices and an increased regulatory or administrative burden for health care workers and managers? And does the term ‘criteria’ (used at the beginning of the process) somehow presupposes a norm which people should follow? These issues were discussed thoroughly and the direction of the project was altered accordingly. Consequently, the term ‘quality criteria of CES’ was changed into ‘quality characteristics of CES’ and it was explicitly stated that the NEON quality characteristics needed to inspire people to improve the quality of CES and not check, certify or judge CES. In this way, in line with a responsive evaluation methodology the stakeholders were enabled to both steer the direction of the network and the results of the process in dialogue.

### Launch of a website and publishing a national handbook on CES

Based on the suggestions and experiences during the first expert meetings (see Organizing expert meetings and national conferences to facilitate a dialogue on quality of CES) a website was launched. On the website (www.hetneon.nl), all people who work in CES can present themselves with a picture, a short biography and contact details. In this way, CES practitioners can make themselves known and find each other. A quarterly newsletter highlighted new content on the website, new NEON participants and other CES related events (e.g., trainings, masterclasses and conferences) and job offers in CES in the Netherlands.

Also participants were invited to publish a practice example on the website to stimulate learning from each other’s practices since this was experienced as very inspirational at the meetings (see Organizing expert meetings and national conferences to facilitate a dialogue on quality of CES). A format was developed in which the participants were asked to answer a set of questions about their own practice example. We asked them: “Please write one or more reasons why this practice example was successful or why it wasn’t successful”. The question of why the practice example maybe wasn’t successful was purposively put in, since less successful CES activities are as informative as best practices of CES (although probably less inspirational). The project group asked to describe pitfalls that would be informative for other CES practitioners and which lessons could be drawn from their experience. Finally, we offered the possibility to attach forms, formats, or documents to the practice examples that can be downloaded from the website. In this way, CES practitioners can share their formats and do not need to reinvent the wheel by developing a form themselves (for instance forms for the evaluation of CES).

Besides a website, also a handbook was mentioned as a desired outcome of NEON at the first expert meeting (see Organizing expert meetings and national conferences to facilitate a dialogue on quality of CES). The handbook was supposed to be a very practical book to support CES practitioners with their CES activities in their health care organization. The content and structure of the handbook was developed in the third expert meeting, while participants jointly made an overview of the chapters of the handbook and the topics to be discussed in each chapter. Five chapters were proposed: 1. reasons for CES; 2. methods for CES; 3. competencies of CES practitioners; 4. implementation of CES; 5. the role of Board and management. Also, participants were invited to indicate in which chapter/topic they wanted to be involved.

Next, drafts of the chapters were written by the first author (LH), based on the information that was collected during the CES activities, the practice examples on the website, the conducted interviews, and the reports of the expert meetings and national conferences. The format of the handbook was meant to be practical and easy to read, providing context, practice examples and thick descriptions of CES, including a ten step plan for implementation of CES and a large number of forms and formats developed by the NEON participants as an appendix. After each paragraph a set of quality characteristics was formulated based as a succinct formulation of the main point about quality of CES in that particular paragraph.

After this, the drafts of the chapters were sent to CES practitioners and participants of NEON who had indicated they wanted to be involved in that chapter. We also purposively approached certain experts in a particular CES activity if we felt we missed the expertise in the group of commenters on a particular chapter (e.g., on the topic of moral counseling and the topic CES in education). We received rich information back in reaction to the drafts, in the form of critical comments, suggestions, remarks, and examples from CES practices. The comments were placed in one document and the project group processed all the remarks.

The handbook was an important step to conceptualize and verbalize the variety of views on the quality of CES. It was emphasized in the handbook that both the content of the book and the specified quality characteristics were not meant as the final and definitive view on the quality of CES. Yet, we did feel the need to summarize all the knowledge we had jointly generated up until then, by stimulating these exchanges and refection about the quality of CES. By publishing them in a handbook, we made the variety of views explicit and summarized the points that CES practitioners agreed upon. In writing the chapters, we encountered a surprising unity in thoughts but also crucial points of divergence in views. For instance, regarding the implementation of CES, some CES practitioners had experienced the importance of support from the board of directors of the health care organization, and stated that without this support you should not even start CES. Other CES practitioners preferred a bottom up approach, in which CES practitioners just start with a micro experiment in one team and then gradually build a program from there. In the handbook, the pros and cons of both approaches were described, without expressing a preference of one over the other; instead, the need to balance these approaches was emphasized.

By co-creating the handbook together with the stakeholders and developing a website on which all the CES practitioners can present themselves, we acted in line with responsive evaluation methodology. Instead of offering abstract theorized knowledge regarding the quality of CES, we focused on unlocking the experiential knowledge of CES practitioners in the field by publishing practice examples on the website and offering contextualized and detailed information in the handbook, in order to inspire and support other CES practitioners.

### Organizing responsive quality assessments

Co-developing quality characteristics of CES was important, yet in the end the question remained how they actually could be used to contribute to fostering the quality of CES in health care organizations. Therefore, in line with responsive evaluation methodology, the meaning and usefulness of the set of quality characteristic was explored within actual CES practices in different health care organizations. The overall objective was again to reflect upon and foster the quality of CES in Dutch health care organizations through a mutual learning process, to stimulate a learning network of CES practitioners, and to evaluate the quality characteristics of CES themselves.

In this phase of the project, 11 health care organizations participated. Due to one withdrawal from the project, 10 health care organizations completed the responsive quality assessment. These health care organizations represented different health care settings. The project included four institutions for people with disabilities, two institutions for people with mental health problems, two academic hospitals, and two general hospitals. The project group used the term ‘responsive quality assessment’ rather than ‘audit’, as the assessment was based on a dialogical, open, responsive way of assessing the quality of CES in line with a responsive evaluation methodology in which CES practitioners themselves reflected upon the quality of ethics support within their own and each other’s health care organizations.

**Figure 1:**
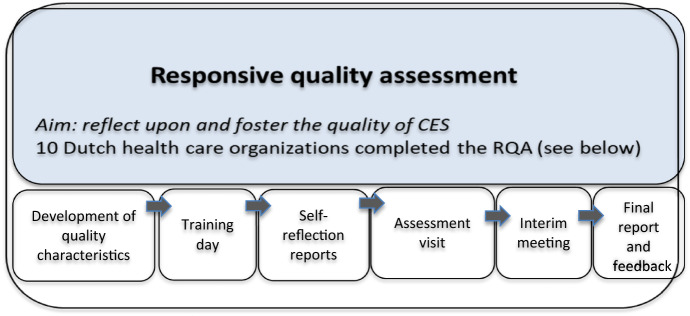
Responsive quality assessments

In the quality assessment, 22 CES practitioners participated. First, we organized a meeting among them to build relationships and to train them in how to responsively evaluate each other’s practices. Before assessing another institution, CES practitioners collected information on CES services within their own organization. In this way, the reflection on their own practice started on the fly, describing their own practice in detail. They were also invited to write a reflection report on CES services within their own organization. This information was sent to the CES practitioners visiting their organization. Subsequently, pairs of CES practitioners from two different organizations would visit a third organization where they would assess the quality of CES. When approximately one-third of the visits had taken place, an interim meeting for all participants was organized by the research team to reflect on the process. After each visit, visiting CES practitioners wrote a report that was shared and discussed with the hosting CES practitioners during a feedback meeting or by email (Figure 1).

Participating CES practitioners had a desire to learn from others and expressed the hope that, through participating in the assessment, their CES work would be acknowledged within their own organizations as relevant to fostering quality of care. The practitioners observed that CES often, yet not always, lacks a formal status within organizations, as well as a substantial budget and clear responsibilities and structure. As a consequence of participating in the responsive quality assessments, respondents perceived a number of improvements regarding CES in Dutch health care organizations: acknowledgement of the relevance of CES; CES practices being formalized; the development of new CES-related activities; and increased reflection on existing CES practices (Van Baarle et al. 2019). Participants were motivated to further professionalize CES services and emphasized the need for a learning community through the Dutch network for CES (NEON).

A limited numbers of CES participants focused in their assessment on the goals and quality of the content of particular CES activities (such as statements about the quality of ethics committee meetings or their policies, advice and letters, or statements about the quality of the MCD facilitator or the arguments within a moral case deliberation). CES practitioners seemed more focused on the implementation of CES, legitimization of its existence, gaining support from upper management and solidifying CES services in their health care organizations than to make concrete what exactly quality of CES at the content level of the CES activities entails. This finding might be related to the fact that CES is still a relatively new field in the Netherlands. Elsewhere, we describe in more detail the challenges and learning experiences of this phase (Van Baarle et al. 2019).

In line with a responsive evaluation methodology, quality assessments by peers of CES practitioners were organized instead of experts assessing the field. Another finding relates to the functionality and usefulness of the CES quality characteristics. Although it was explicitly and repeatedly mentioned that the quality characteristics were not meant as an objective measurement but intended to inspire, some participants continued to interpret them as objectively prescribing “the right CES”, or as intimidating and even demotivating in the sense of “We can never live up to this full list of quality characteristics, why should we even start?”. To address this, we changed the tone of voice, and summarized and shortened the list of quality characteristics, emphasizing that the quality characteristics are meant as a heuristic instrument, stimulating a dialogue and refection on the quality of CES (Hartman et al. In Press).

## Discussion

In this paper, we described the development of the Dutch network for ethics support (NEON) and reflected on our methodology to conceptualize and foster the quality of CES. In line with our theoretical understanding of CES, our activities were inspired by a responsive evaluation methodology. Within CES one deliberates about what is morally good, how one can determine this and which presuppositions underlie one’s views and arguments. Inspired by philosophical theories, such as pragmatism and hermeneutics, we presuppose that moral good is co-created with the stakeholders involved in a specific moral issue (Hartman et al. 2020). Similar to this understanding of ethics and practicing CES, we argued that the norms and criteria for determining what a good quality of CES also need to be formulated through a joint reflection process by the stakeholders about their underlying presuppositions about the quality of CES, based on concrete experiences.

Instead of setting standards first and then trying to move CES practices towards these standards, a responsive evaluation methodology allows fostering of the quality of CES practices by encouraging CES practitioners to reflect on the standards they themselves and others hold and at the same time jointly define what this quality actually entails. For this process, co-ownership of both the process and the outcome is crucial. Also, active participation of the stakeholders is important for the process. In the following, we discuss three distinctive features of our approach and reflect on the differences and similarities with other national networks.

First, NEON includes all types of health care (not only hospital care as is the case for the ASBH and UKCEN) and all types of CES (not only clinical ethics committees, as is the case for UKCEN or ethics consultation as is the case for the ASBH) (ASBH 2011; Slowther 2008). This way, different types of CES can learn from each other and inspire one another. This approach is possible, since the Netherlands is a relatively small country, making the numbers of CES practitioners relatively low. Also, NEON has, until now, explicitly distanced itself from a certification program, based on input from the NEON participants, different from the ASBH (ASBH 2020b). This is in line with a responsive evaluation methodology in which the stakeholders are encouraged to steer both the direction and the form of the evaluation process itself.

Second, our approach aims at unlocking experiential knowledge in the form of sharing practice examples of CES on the website and in the handbook. For this aim, fostering and creating new relations between CES practitioners and other stakeholders in order to be able to share these experiences is of great importance. This is also resembled in the way the NEON website is designed. The website of NEON differs from, for instance, that of UKCEN or the ASBH (UKCEN 2020; ASBH 2020a). Whereas the website of UKCEN contains educational resources, and the website of ASBH provides documents about standards, the website of NEON focuses specifically on knowledge exchange, concrete practice examples and CES practitioners presenting themselves to each other (NEON 2020). This reflects our theoretical understanding of CES as well as responsive evaluation methodology, as both emphasize that experiential experience is an important source for knowledge, contextual details are relevant to asses a situation and deliberation between multiple perspectives is crucial to assess a situation (whether it is assessing what is the right thing to do in a moral issue, or assessing what is a good quality CES activity).

Third, we focus on an ongoing process of learning together with stakeholders instead of providing norms on CES and assessing whether a certain CES activity meets that norm. Whereas other national initiatives have presented drafts of quality documents regarding CES to stakeholders, we tried to involve the stakeholders more fundamentally, for instance, by also discussing the process with them, similar to the methodology developed in Canada (Kirby and Simpson 2012). Moreover, besides developing the CES quality characteristics together, we also applied the quality characteristics in responsive quality assessments in which CES practitioners themselves visited each other to start a dialogue about the quality of CES in their health care organizations (Van Baarle et al. 2019). This again provides an example of joint learning. The quality characteristics are meant to function as a heuristic instrument, and will be continuously evaluated and updated based on the experiences within CES practices (Hartman et al. In Press).

These distinctive features of our approach may clarify what a responsive evaluation methodology has to offer. Responsive evaluation can be viewed as collaborative, participative, and capable of generating change. This does not mean that other approaches are not useful. Guidelines or sets of values may inspire CES practitioners to look at other themes that may also be relevant for fostering the quality of CES services and provide CES practitioners with clarity and stability. Thus, a diversity of approaches to conceptualize and foster the quality of CES is important as they can reinforce each other. In order to better understand the strengths and weaknesses of each of these approaches, we argue that it is important to be explicit about one’s normative presuppositions and to study how and in which way these approaches contribute to the quality of CES. This can help the international debate forward and help to professionalize the field of CES (Schildmann et al. 2013).

## Conclusion

In this paper, we described the development of NEON, a national network for CES in the Netherlands, which aims to define and foster the quality of CES. Within NEON, we organized activities inspired by a responsive evaluation methodology, as this methodology bears close resemblance with our theoretical understanding of CES, which is based upon philosophical theories, especially hermeneutics and pragmatism. We elaborated on three central activities we undertook and we reflect upon how these activities contributed to the conceptualization and fostering of the quality of CES in the Netherlands: 1. Organizing expert meetings and national conferences to facilitate a dialogue on quality of CES. 2. Launch of a website and publishing a handbook on CES. And, 3. organizing responsive quality assessments. Our approach is different from other national approaches in three ways: a broad inclusion of different health care domains and different types of CES; a focus on experiential knowledge and fostering relations between stakeholders; and a focus on an ongoing joint learning process instead of providing knowledge and/or norms as a final product.

NEON aims to encourage reflection and dialogue between CES practitioners, resulting in a full and broad understanding of several elements of the quality of CES. This is an ongoing process. The quality of CES is dependent on the views, perspectives, knowledge and insights of all participants. So, we will need to continuously update our views, and reflect on new developments. NEON can play a role in this process, and will hopefully contribute to the further dialogue on the quality of CES in the Netherlands and evaluate the developed quality characteristics and other quality related products of NEON.

## Data Availability

Since this is a more theoretical oriented paper, no additional data are available in a database. Supporting documents from the research activities we could be made available upon request (in Dutch).

## Footnotes

1 Also in other countries, fostering the quality of CES is on the agenda, but because of limited space we do not describe these activities in detail. See respectively for Germany, Switzerland, Italy, Japan and Israel: (Schochow et al. 2019; Reiter-Theil 2008; Caminiti et al. 2011; Akabayashi et al. 2008; Wenger et al. 2002). There is also a European Clinical Ethics Network (ECEN), founded in 2005. ECEN aims to improve the quality and professionalism within the field of clinical ethics support in Europe (Pedersen et al. 2010).

2 Over 1400 copies of a draft of the text were distributed – the taskforce received over 200 comments. In the sixth meetings, they incorporated these comments and established the final version.

3 The taskforce received 60 reactions. The document was rated by “55% percent of the respondents as “excellent”, 33.3% as “good”, 10% as “fair” and 1.7% as “poor”. See note 10, ASBH 2011: p.56.

4 The VA is the largest integrated health care system in the U.S. providing care at 1,255 health care facilities; including both 170 Medical Centers and 1,074 outpatient sites of care.

5 In 2013, PHEEP continued in the network CAPHE-ACESS (Canadian Association of Practicing Healthcare Ethicists – Association canadienne des éthiciens en soins de santé). Since CAPHE-ACESS/PHEEP is mainly focused on hospital care (like UKCEN), another network has been set up in Canada that focuses on community care, called CEN (Community Ethics Network (CEN). CEN is a membership-funded collective of over 50 organizations with the mission to lead and sustain the integration of ethics within and across community health and support sector. As a paying member one can access a community ethics toolkit, including a code of ethics and an Ethical Decision-Making Worksheet, at their website. https://communityethicsnetwork.ca/

6 Deliberative engagement is fostered by four elements: A. skilled facilitation; B. adoption of an “engaged participation” role; C. use of interactive elements of deliberative dialogue; and, finally, D. democratic, consensus-seeking decision making.

7 Core stakeholders are defined as the national community of Canadian Practicing Health care ethicists.

8 Secondary stakeholders are divided in tiers: tier one consist of those with whom practicing health care ethicists directly work; tier two consists of primary academics, research ethics board practitioners, employers, related professionals, international ethics associations and research funders, and finally tier three consist of members of the public-at-large.

9 With a carrousel, we mean a work form in which the group was divided in smaller groups. These smaller groups “rotated” in the room, brainstorming simultaneously about these different questions at designated tables and, after a dedicated amount of time, moved towards the next table and question.
